# Modulation of neural responses in inferotemporal cortex during the interpretation of ambiguous photographs

**DOI:** 10.1111/j.1460-9568.2008.06263.x

**Published:** 2008-06

**Authors:** Yan Liu, Bharathi Jagadeesh

**Affiliations:** Department of Physiology & Biophysics, University of WashingtonSeattle, WA 98195, USA

**Keywords:** attention, classification, macaque, object recognition

## Abstract

Ambiguous images are interpreted in the context of biases about what they might be; these biases and the behavioral consequences induced by them may influence the processing of images. In this report, we examine neural responses in inferotemporal cortex (IT) during the interpretation of ambiguous photographs created by morphing between two photographs. Monkeys classified different images as being one of two choices and learned to classify most of the samples correctly. For one image (the ambiguous sample) reward was administered randomly for either possible choice, and the monkeys were free to classify that image based on their own interpretation, with no learning possible. The ambiguous samples were not classified randomly: the monkey interpreted the samples differently during different sessions. The interpretation of the ambiguous sample was, in turn, highly correlated with the normalized response of individual neurons in IT to the ambiguous sample. If an ambiguous sample was interpreted as a particular choice during a session, the response to that ambiguous sample more closely resembled the response to that choice. Identical ambiguous images were interpreted differently during different sessions, and neural responses reflected the differing interpretations of the image during that session. The relationship between the interpretation of the image and neural responses strengthened over the course of a session because neural responses shifted to more closely resemble the response to the initial interpretation of the image. The data support a flexible representation of visual stimuli in higher visual areas.

## Introduction

The visual world is ambiguous and cluttered and cannot be precisely stored in our brains. Our brains do not have a ‘photographic’ ability to store or recall visual images and scenes. Instead, the brain does both more and less, imposing structure onto ambiguity and discarding information that contradicts the imposed structure (Gilbert & Sigman, 2007). This process is particularly important when viewing ambiguous or conflicting visual information (Bruner & Potter, 1964; Bar & Aminoff, 2003; Leopold, 2003; Bar *et al.*, 2006; Bar, 2007).

Human studies suggest that expectations and assumptions about the content of the ambiguous images can influence the perception of the image, and can alter neural responses to images in sensory areas (Bruner & Potter, 1964; Dolan *et al.*, 1997; Bar & Aminoff, 2003; Ranganath *et al.*, 2004a; Kamitani & Tong, 2005; Ranganath & D’Esposito, 2005; Rotshtein *et al.*, 2005; Sergent *et al.*, 2005; Bar *et al.*, 2006; Bar, 2007; Del Cul *et al.*, 2007; Furl *et al.*, 2007a; Kveraga *et al.*, 2007; Li *et al.*, 2007). An interactive network consisting of multiple brain areas may resolve ambiguity in images within psychological and perceptual contexts (Li *et al.*, 2007; Sterzer & Kleinschmidt, 2007).

The sensory cortex in which these influences play out include higher order visual areas such as inferotemporal cortex (IT) (Bar & Biederman, 1999), where cells are sensitive to the features contained in complex images (Desimone *et al.*, 1984; Kobatake & Tanaka, 1994; Tanaka, 1996; Allred *et al.*, 2005; Hung *et al.*, 2005; Kiani *et al.*, 2007). Neurons in the IT are sensitive to changes in the percept of an ambiguous image during binocular rivalry (Sheinberg & Logothetis, 1997) and during the perception of ambiguous disparity (Uka *et al.*, 2005). Furthermore, neural responses to images are modulated by the demands of tasks, learning and attention (Moran & Desimone, 1985; Miyashita, 1988; Desimone & Duncan, 1995; Tovee *et al.*, 1996; Erickson & Desimone, 1999; Erickson *et al.*, 1999, 2000; Jagadeesh *et al.*, 2001; Messinger *et al.*, 2001; Sigala & Logothetis, 2002; Kourtzi & DiCarlo, 2006; Mirabella *et al.*, 2007). IT is therefore likely to show the influences of stimulus interpretation. In this report, we examine the properties of neuronal responses in IT during the interpretation of ambiguous stimuli with the goal of examining the relationship between the interpretation of the images and the responses in IT. We recorded from IT neurons during the classification of ambiguous morphed photographs. One stimulus created by the morphing was ambiguous, both visually and behaviorally. We recorded neural responses in IT while the monkeys classified the ambiguous image (and other, non-ambiguous, sample images) in a two-alternative delayed match-to-sample task. Under these circumstances, across sessions and stimuli, there was substantial variability in the classification of the ambiguous sample (AS), indicating variability in the interpretation of the AS image. Across sessions, the normalized response of individual cells to the AS correlated well with the average probability of choosing one of the choice stimuli. The correlation was seen for identical AS images which were interpreted differently during different sessions. The correlation increased over the course of the recording session as, over the course of the session, responses to the AS image became aligned with the interpretation of the AS. No change in behavior (learning) was expected in this task as choices with the AS were rewarded randomly, and none was seen. Thus, the change in neural response occurred in the absence of any compatible shift in behavior with the AS over the course of the session. These data are compatible with a schema of IT in which the interpretation of ambiguous images alters the representation of the image so that neural responses come to match the interpretation of the image. The mechanism for modulating the neural responses could be experience-dependent (but not learning-dependent) modification of the neural responses (Messinger *et al.*, 2005) or consistent modulation by attention (Maunsell & Treue, 2006; Nobre *et al.*, 2006). The findings provide evidence for a flexible mapping of visual input to neural responses in IT; this flexibility could be exploited to produce a match between the perception of images and neural responses in IT (Allred & Jagadeesh, 2007; Liu & Jagadeesh, 2008).

## Methods

### Experimental methods

#### Surgery

Two male adult monkeys (Macacca Mulatta) weighing 5–8 kg were used in these experiments. Standard techniques were used for recording from awake behaving primates (Allred *et al.*, 2005): surgery on each animal was performed (under gaseous isoflurane anesthesia) to implant a head restraint, a cylinder to allow neural recording and a scleral search coil to monitor eye position (DNI, Newark, DE, USA; Judge *et al.*, 1980). Eye movements were monitored at 500 Hz. The cylinder was implanted using stereotaxic measurements to access IT (described below). All animal handling, care and surgical procedures were performed in accordance with guidelines established by the NIH and approved by the Institutional Animal Care and Use Committee (IACUC) at the University of Washington.

#### Recording procedures

Single-unit recordings were made using standard techniques. An *x-y* stage for positioning and an electrode holder containing a sterile guide tube and tungsten microelectrode (Alpha-Omega, Nazareth, Israel) were attached to the top of the recording cylinder. The guide tube was lowered to ∼15 mm above the expected location of IT, using stereotaxic coordinates, and the electrode was moved using a microdrive (David Kopf Instruments, Tujunga, CA, USA) and signals from the electrode were sorted online using the Alpha-Omega spike sorter. Responses of single IT neurons were collected while monkeys viewed images and performed the two-alternative forced-choice–delayed match-to-sample (2AFC-DMS) task described below. Coded spikes were stored on a PC at a rate of 1000 Hz using CORTEX, a program for neural data collection and analysis developed at the NIH (Bethesda, MD, USA). Online histograms were created to qualitatively judge selectivity, but for the purposes of this paper all data analysis was performed *post hoc* on stored data. Materials for these procedures were obtained from Crist Instruments (Hagerstown, MD, USA) and DNI.

**Fig. 1:**
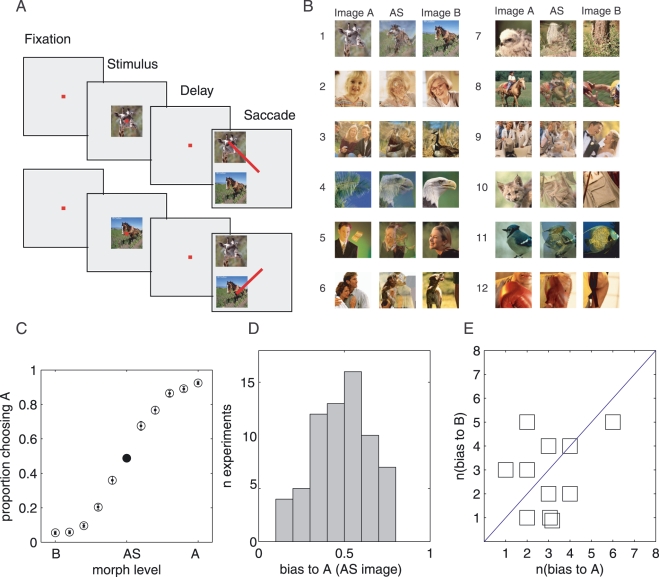
Task and behavioral performance. (A) 2AFC-DMS task with two possible sample stimuli appearing on different trials. Samples could be one of the pair of photographic images (which also served as the choice array) or any of 11 possible morphed stimuli. One example image pair is shown, but the images used could be any of 11 other pairs. (B) Images used as samples: AS created by morphing or interpolating between the two original photographs, image A and image B, averaged across sessions (*n* = 67). (C) Proportion of selections of Stimulus A as a function of the similarity to image A. (D) Distribution of behavioral bias towards A across sessions (*n* = 67). (E) Number of sessions in which individual stimuli resulted in a bias towards A vs. number of sessions in which individual stimuli resulted in a bias towards B (*n* = 11 images; image pair no. 2 was excluded because no selective cells were found for that image pair).

Neurons were selected using anatomical and physiological criteria. Structural MRI was used to guide placement of the recording chambers, which were centered in stereotaxic coordinates 16–17 mm lateral and 18–22 mm anterior, over the right hemisphere. Neural recordings were targeted to the center of the chamber, near the perirhinal sulcus and the anterior middle temporal sulcus. However, the selection criterion for recording locations was the presence of cells that responded selectively to one of the 12 image pairs used in this study, and recording locations were altered until such selectivity was found. We sampled locations within the chamber, moving 0.5–1 mm when appropriate selectivity for the images was not found for 2–3 days. When selective cells were found, the area was re-sampled in the same track until we could no longer isolate cells with selectivity for one of 12 pairs of images (Fig. 1B). Anatomical data is unavailable for the precise locations of recording sites because animals are still being used in related experiments. The experimenter usually found an apparently selective neuron (one included in the population presented in this report) after sampling one to three sites in a session. Upon isolation of an apparently selective neuron, the monkey began performing the 2AFC-DMS task with the stimulus set for which the isolated neuron was selective.

### Behavioral paradigm and data collection

#### 2AFC-DMS task

The monkey performed the 2AFC-DMS task (Fig. 1A) with two different sample stimuli (chosen from one of 12 pairs of photographic images; Fig. 1B) and the nine morphed variants of those two samples (for description of morphing, see below and Liu & Jagadeesh, 2008). The target choices always consisted of the pair of photographic images, the choice images; the monkey was required to classify each of the morph variants as one of the pair by making a saccade to the matching stimulus in the choice array, based on the similarity of the sample to one of the two choices.

An example image pair and associated trials are illustrated in Fig. 1A. Each trial began with the presentation of a fixation point (0.3°, red square). After the monkey acquired fixation (within a 4°-diameter window centered on the fixation spot; the window was matched to the size of the sample image), there was a variable delay before the onset of the sample, presented for 320 ms. After another variable delay period (mean 912 ms, 700–1100 ms), the choice array was presented. The two choice stimuli were located 5° to the left, and either 5° up or down from the fixation point. Location of the two choices was randomized between the two positions, so the monkey could not determine the location of the correct saccade before choice array onset. All images (both samples and choices) subtended 90 × 90 pixels, which was ∼4° of visual angle at normal viewing distance. After another variable delay period (mean 567 ms, 412–1212 ms), the fixation point turned off, providing a cue that reward was available for making a saccade to the correct stimulus in the choice array. The monkey's task was to make a saccade to the choice that matched the stimulus, and maintain fixation on that choice for 500 ms. If the monkey initiated a saccade before the cue, the saccade choice was recorded; then both target images were turned off, and no reward was administered. When a saccade occurred after the cue, and a correct saccade was made, the monkey received a reward.

Analysis of latency from choice array onset to saccade onset indicated that, rather than using the cue to guide behavior on a trial-by-trial basis, both monkeys made their saccades at a stereotyped latency after the onset of the choice array, centered on the mean time of cue (mean cue of reward availability 558 ms; mean latency 860 ms); the monkeys did not learn the relevance of the cue on each trial, and instead waited long enough to obtain reward on most (93%) of the correct trials. All trials in which the monkey made a choice from the choice array were included in this analysis; trials in which the monkey aborted the trial before a choice occurred are not included.

In a single session or block of trials, the monkey performed the 2AFC-DMS task with eleven possible sample images [one pair of photographic images chosen from a set of 12 possible image pairs (Fig. 1B, labeled A and B) and nine morphed variants, described below] presented randomly within a block of trials.

#### Eye movements

Monkeys were free to make saccades within the 4°-diameter fixation window during the period surrounding the presentation of the sample and before making their choice from the choice array. However, 90% of fixations were contained within a 1° window centered on fixation. There were no detectable systematic differences in the position of the eyes during the presentation of different stimuli or during different sessions. No changes in eye position were detectable over the course of a session.

#### Fixation task (search)

The monkeys performed a fixation task while the experimenter isolated an appropriate cell for performance of the 2AFC-DMS task. In this task, the monkey was rewarded for maintaining fixation within a 4°-diameter window while two successive identical images were presented for 300 ms, separated by a 300 ms interval. Stimulus sets for the search fixation task consisted of the 24 images shown in Fig. 1B, labeled A and B (left and right columns, Fig. 1B). The fixation task was used solely to search for selective cells. Using this task, the experimenter chose one of the 12 pairs of photographic images (Fig. 1B) to use in the 2AFC-DMS task and the morph fixation task described above. The pair was chosen when qualitative assessment of the isolated neuron suggested that the recorded neuron produced stronger responses to one image in the pair than to the other image; too few trials were collected in this task to characterize the neural responses to the 24 images, and the task was used only to search for cells.

#### Animal training

The animals were trained using operant conditioning techniques with water or juice as the reward for desired behavior. Each monkey was trained first to complete the 2AFC-DMS task with one sample image, which remained consistent throughout a block of trials. When the monkey was consistently picking that sample image from the choice array, the alternative sample image (the other image in the pair) was presented as a sample image while the monkey learned to choose the matching image from the choice array. After the monkey performed well in this ‘reversal’ training, we decreased the number of trials in blocks in which one sample images was presented. Once blocks were < 10 trials in length, we randomized the sample presentations so either sample stimulus could appear any trial. When the monkey performed > 90% with two sample stimuli, we introduced the morphed sample variants, first including the eight additional sample variants for which there was a correct response, and finally introducing one additional sample variant which putatively consisted of equal parts of the pair of photographic images, and for which choices made for that image were rewarded randomly (the AS). Monkeys were trained extensively with the 12 image pairs and their morphed variants before recording sessions began. Thus, both the original images and the morphed variants were familiar to the monkeys before the neuronal data were collected.

#### Morphed images

Each of the 12 pairs of images was morphed using MorphX (http://www.norrkross.com/software/morphx/morphx.php), a freeware, open-source program for morphing between two photographic images. Details of the algorithm are available from the source code for the MorphX. A brief description of the morphing process is included here: first, the experimenter chose two images (image A and Image B, Fig. 1B); then she set control points on the two images to designate corresponding areas. MorphX then created a series of image frames warping the surface of image A to the control points on image B, simultaneously altering image colors in image A to correspond to image colors in image B. Nine intermediate images in sequence between the two original images were created.

One image, the AS, is putatively located at the midpoint between the two images in the choice pair on the morphing continuum; this image was putatively equally similar to the two original images from which it was morphed. The perceptual similarity of the AS to the two originals cannot be determined quantitatively, but choices with this stimulus were rewarded randomly and, thus, behavior with this image was ambiguously reinforced (see below).

#### Reward contingencies for morphed images

The morphed variants, along with the individual images in the choice pair, were used as samples in the 2AFC-DMS task described above. In every case, the monkey's assigned task was to classify the sample image as one image in the choice pair (the images from which the samples were morphed), by judging the similarity between the image presented during the sample period and the two available choices. The monkey was rewarded for a correct classification when the morphed sample was located closer in the morph sequence to the image. The monkey was rewarded randomly for the one morph variant that was putatively at the midpoint of the sequence, the AS, described above. Reward for all other images was available when the monkey chose the choice image that was more similar to the sample image (for the eight other images, four similar to Image A and four similar to Image B). The data discussed in this report primarily concern the AS image, and the response to the two original photographs from which the AS was morphed. A discussion of the responses to the non-ambiguous images and behavior with them is included in Liu & Jagadeesh (2008). The AS image was over-sampled compared to the two original images: the number of trials of the AS image ranged from 19 to 51 trials, mean ± SD 34 ± 7 trials. The two original photographs were presented on average 16 ± 4 trials each.

#### Effective (Eff) and ineffective (Ineff) stimuli

In characterizing the neural and behavioral performance, the two original photographs were termed the Eff and Ineff image, based on the responses during the sample period to those two images; by definition the Eff image produced a larger response than the Ineff image. Either of the two original images could be the Eff or Ineff image, and neither image was more often the Eff image across cells (*P* = 0.0662, χ^2^ test).

#### Neuronal database (2AFC-DMS task)

Selection of cells for inclusion in the population could influence the conclusions made in this report. We therefore detail the selection of cells for the neuronal database in this section. We attempted 163 recording sessions in which the monkey performed the DMS task. In 123 (75%) of them, we were able to successfully isolate cells that appeared to be selective for one of the 12 image pairs (using qualitative criteria); the total number of sessions include ones in which the experiment was unsuccessful for reasons other than finding selective neurons (e.g., broken electrodes, poorly performing monkey, etc.); therefore, the real frequency of finding an appropriate cell to include in the population is underestimated. Typically, one or two sites were sampled before including a cell in the population. Thus, the cells described in this report were common (found after sampling one or two sites, in 75% of attempted sessions). One hundred and fifty-four experiments were carried out in the 123 sessions (some sessions resulted in more than one cell and some cells were recorded with more than one image set). Cells were pruned from the data set for two behavioral criteria: (i) performance < 60% for all three of the easiest discriminations (morph levels 3–5; seven experiments); (ii) less than five trials of data collected for each sample stimulus (six experiments). Excluding these experiments resulted in 141 data sets. We then selected visually selective cells (significant differences between the Eff and Ineff stimulus for the original images or closely related morph variants, *P* < 0.01 for at least 2/3 of the 3 images most similar to the Eff and Ineff image) for further analysis. This criterion resulted in a remaining population of 67 experiments, consisting of cells that were highly selective for the Eff vs. Ineff stimulus.

#### Analysis of neural data

Neural data were analyzed during the presentation of the sample image, and the period immediately after the presentation of the sample image and before the presentation of the choice images. Mean responses were calculated in epochs following the onset of the sample image, and peristimulus time histograms (PSTHs) are shown for the period after the onset of the sample image. Responses during the presentation of the choice images are not included in this report.

#### Similarity index (SI)

An SI for the AS was calculated for each neuron, corresponding to the equation below:
(1)
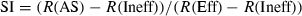

where R(AS), R(Ineff) and R(Eff) are the mean responses to the AS and the Ineff and Eff stimuli, respectively, in the chosen epoch. The epoch used was usually 75–375 ms after sample onset, and is referred to as the sample epoch. The SI is 0 when the response to the AS equals the response to the Ineff image, and 1 when the response to the AS equals the response to the Eff image; it can range below zero and above one if the response is slightly bigger or less than the Eff and Ineff image. The same value can be created for images other than the AS, substituting the response to an alternative image to the R(AS).

PSTHs, time-locked to sample onset, were normalized when averaging across cells using an analogous index:
(2)
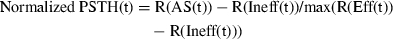

where R(AS(t)), R(Eff(t)) and R(Ineff(t)) are mean responses as a function of time to the AS, Eff and Ineff stimuli, respectively.

#### Response changes over session

To examine response changes over the course of a session, we divided the responses to the AS, the Eff and Ineff stimuli into four quartiles based on the number of trials of those stimuli in the entire session. Each quartile contained 1/4 of the trials for that particular stimulus. We then calculated the SI and normalized PSTHs for each quartile separately. The precise proportion of the trials (1/2 or 1/3 or 1/5) included in each block did not change the overall results.

#### Choice probability

We calculated choice probability by dividing the responses to the AS image for an individual neuron into two groups of trials based on the choice made with that AS image on that trial. We then compared the responses in the two groups using the receiver operating curve (ROC) statistic, comparing the response in trials in which the monkey chose the Eff image to trials in which the monkey chose the Ineff image, calculating the area under the ROC curve (Green & Swets, 1966), resulting in a calculation of choice probability in selected response epochs (Britten *et al.*, 1996). Choice probability was calculated in an epoch before the onset of the stimulus (baseline epoch, −400 to −100 ms before sample onset), a post-sample epoch (200–1000 ms after sample onset) and in three divisions of the post-sample epoch (75–375 ms after sample onset, 375–675 ms after sample onset and 675–975 ms after sample onset).

## Results

We recorded neural activity in anterior IT during the interpretation of ambiguous photographs. The monkeys performed a 2AFC-DMS task in which they were asked to classify a sample image as one of two possible choices by making a saccade to it (Fig. 1A). We recorded 154 neurons in 123 separate sessions during performance of the task using 12 different possible image sets (Fig. 1B). Sixty-seven of these neurons were significantly selective for the pair of choice images used in the recording session, when the choice images were presented as the samples, and this report examines the relationship of those neurons to the interpretation of the ambiguous image.

### Behavior

Monkeys learned to do the 2AFC-DMS task well, as demonstrated by their performance in matching the original sample images to the identical choice images: classifying image A correctly, 0.9245 ± 0.0102; classifying image B correctly, 0.9460 ± 0.0101. Figure 1C shows the proportion of choices of stimulus A for each of the eleven different possible morphed sample images, ranked by their similarity to the original images. Performance was high for the original images (A and B), and behavior changed systematically as a function of the similarity of the individual sample images to the originals (Fig. 1C).

In the context of this excellent performance with most of the sample images, the interpretation of the AS was quite variable. Across sessions, monkeys were equally likely to classify the AS as either stimulus choice (image A or image B; bias to A, *n* = 33; bias to B, *n* = 31; *P* = 0.82, χ^2^ test). However, between sessions there was substantial variability in the classification the AS, ranging from 0.12 to 0.88 choices of stimulus A, despite the fact that the monkeys were rewarded randomly for choices in each of those sessions (Fig. 1D). The same AS image could be classified differently during different sessions and the number of sessions in which each of the AS images was classified more frequently as stimulus A (Fig. 1E; *x* axis, *n* of *P*(A) > 0.5) or more frequently as stimulus B (Fig. 1E; *y* axis, *n* of *P*(A) < 0.5) was balanced (*P* > 0.40, χ^2^ test, for each of the eleven images).

### Neural response

**Fig. 2:**
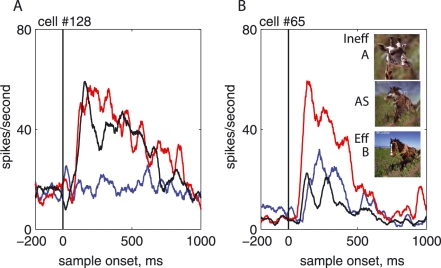
PSTHs for two different experiments with the same images (the horse/giraffe pair). Time 0 was the onset of the sample; sample duration was 320 ms. Red line, Eff image (Horse, image B); blue line, Ineff image (Giraffe, image A); black line, AS (horse/giraffe). (A) Session in which monkey was biased towards classifying the AS as the Eff image, or the Horse [proportion bias to Eff image (image B) = 0.81, SI = 0.79]. (B) Session in which monkey was biased towards classifying the AS as the Ineff image, or giraffe [proportion bias to Eff (image B) = 0.28, SI = −0.27].

Is this variability in classification (i.e. interpretation of the image) correlated with the neural response to the AS during the sessions? Two representative cells in Fig. 2A and B were found in two different sessions, in which the monkey classified the same AS image (the horse/giraffe, image #1, Fig. 1B) differently (A, AS interpreted as horse; B, AS interpreted as giraffe). The histograms show the mean response across each session to the two original photographs (horse, red; giraffe, blue; horse/giraffe, AS, black). The response for both cells was bigger for the image of the horse than for the image of the giraffe. In order to describe behavior in terms compatible with neural activity, we defined an Eff stimulus for each cell (the image that produced the larger responses is the Eff, the other is the Ineff image) and describe the behavior with respect to the Eff and Ineff stimuli. For these two cells the Eff images were identical but, across cells, either image A or image B in a pair of images could be the Eff stimulus for a cell, and neither image was significantly more likely to be the Eff image (A = Eff, *n* = 44; B = Eff, *n* = 23; *P* = 0.0662, χ^2^ test).

These two cells were recorded in different sessions in which the monkey's behavior with the AS was different (Fig. 2A, p(horse, Eff) = 0.81; Fig. 2B, p(horse, Eff) = 0.28). Thus, in this instance the same image (the horse giraffe) was interpreted differently in the two sessions, countering the possibility that each AS image was always classified in the same way. Inspection of the response histograms further shows that these two different example neurons exhibited a different response to the identical AS image in the two sessions. For the cell in Fig. 2A, the response to the AS resembled the response to the Eff image while, in the cell in Fig. 2B, the response to the AS resembled the response to the Ineff image. Individual neurons, of course, could show differences in neural responses to the identical AS, purely because the two neurons were selective for different features in the AS that have differing relationships to features in the Eff and Ineff stimulus. In these examples, however, the response differences to the AS also mapped to the behavioral difference in the two sessions. The response to the AS was higher, and resembled the response to the Eff stimulus (the horse), when the AS was frequently classified as the Eff stimulus (Fig. 2A) while the response to the AS was lower, and resembled the response to the Ineff stimulus (the giraffe), when the AS was frequently classified as the Ineff stimulus (Fig. 2B). To quantify the similarity of neural responses of the AS to the responses to the Eff an Ineff, we calculated an SI (see Methods) using an epoch 75–375 ms after the stimulus onset. The SI normalizes the response of the AS image for each cell to the response range of the cell (the difference between responses to the Eff and Ineff images). For these two cells, the SI was 0.79 for the cell in the session in Fig. 2A (bias to Eff, 0.81) and −0.27 for the session in Fig. 2B (bias to Eff, 0.28). The SI was higher (similarity of response to the Eff was higher) when the monkey more frequently classified the AS as the Eff stimulus, and the SI was lower when the monkey more frequently classified the AS as the Ineff stimulus.

**Fig. 3:**
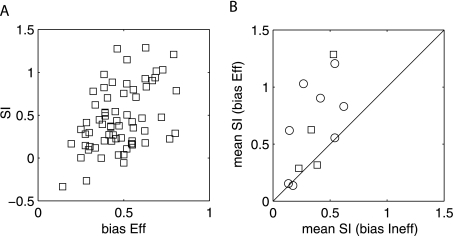
(A) SI vs. the bias in classifying the AS as the Eff image; *r* = 0.45, *P* = 0.0001, *n* = 67. (B) Mean SI for identical AS images, in sessions in which the average bias was towards the Eff image (*y*-axis) vs. sessions in which the mean bias was towards the Ineff image (*x*-axis). Each point is a different image from the set of images in Fig. 1B (*N* = 8 + 4). The mean SI was greater for each image when the behavior was biased to the Eff image than to the Ineff image (paired *t*-test across images: *P* = 0.0067, *n* images = 12, *n* experiments = 44).

This relationship was found across the entire population of cells selective for the photographic images. The relationship between the SI index and the proportion of classifications of the Eff stimulus across the population of cells (Fig. 3A) shows a robust correlation between the SI of individual neurons and the classification of the AS image (*r* = 0.45, *P* = 0.0001, *n* = 67). The correlation shows that the monkeys classified the AS as the Eff stimulus more frequently in sessions in which the recorded cells’ response to that stimulus more closely resembled the response to the Eff image.

### Classification of identical AS images

In the examples shown in Fig. 2, identical images were sometimes interpreted differently in different sessions, and the responses recorded at the same time resembled the interpretation during these different sessions. If this characteristic is a general property, the response difference produced on different interpretations can be separated from differences based on the stimulus itself. To examine whether the property seen was general, we examined the neural responses of all cells to AS images in sessions in which the same image was classified differently. We found all of the AS images that were classified differently during different sessions and plotted average SI in sessions in which the interpretation was towards the Eff stimulus against the SI sessions in which the same AS resulted in an interpretation to the Ineff stimulus (Fig. 3B). The responses in the two groups of sessions were significantly different across the stimuli [mean SI(interpretation to Eff) = 0.565; mean SI(interpretation to Ineff) = 0.387; paired *t*-test, *P* = 0.0067].

### Trends in neural responses and behavior

**Fig. 4:**
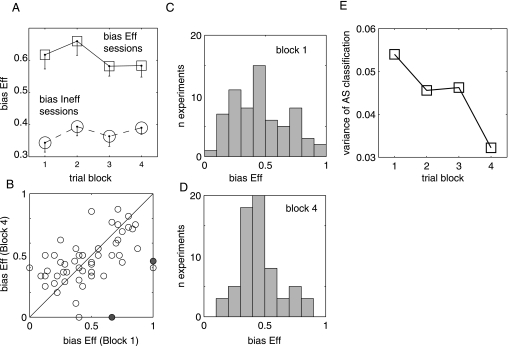
(A) Mean bias (SI), Eff bias (squares) and Ineff bias (circles) sessions as a function of block in the session. (B) Bias during block 4 of session plotted against bias during block 1 of the session. (C) Distribution of bias in block 1 of the session. (D) Distribution of bias in block 4 of the session. (E)Variance (SD^2^) of classification bias as a function of block.

The differences in behavior (and neural response) during different sessions with identical stimuli raised the possibility that behavior and neural responses might also change during a single session. To address this possibility, we analyzed trends in behavior and neural response over the course of a single session by dividing each session into four quartiles. First, we examined the behavior with the AS image in each of these individual blocks (Fig. 4), broken down by the overall behavior over the course of the entire session: the comparison that yielded a differences in neural response over different sessions (Fig. 3B). These two groups of sessions showed no detectable trends over the course of the session (Fig. 4A; two-way anova, *P* > 0.10, interaction and effect of block). The bias in block 4 was also compared to the Bias in block 1 (Fig. 4B). Significant scatter is visible but no consistent trends. In only two of these sessions was the bias seen in block 4 significantly different from the bias seen in block 1 (*P* < 0.05, χ^2^ test, marked in grey). In the scatter graph, there is a weak trend towards high bias sessions having slightly higher bias in block 1 than in block 4 (and the reverse for low bias sessions). When broken down by block, this weak trend was seen as a decrease in the variance of behavior found in the first block compared to that in the fourth block (Fig. 4C–E).

**Fig. 5:**
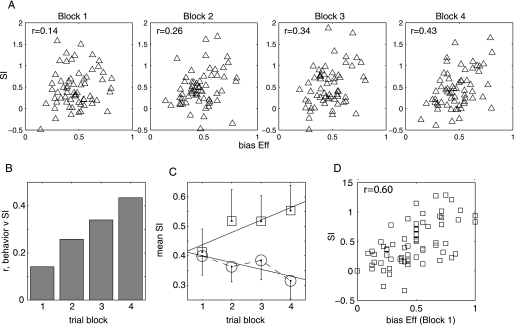
(A) SI vs. the bias in classifying the AS as the Eff image in four successive 1/4 block of trials in the session. (B) *R*-value of correlation between SI and classification bias towards A as a function of block in the session. (C) Mean SI in the same Eff bias (squares) and Ineff bias (circles) sessions as a function of block in the session. (D) SI vs. bias in classification to Eff in block 1; *r* = 0.6013, *P* < 0.00001).

The behavior showed no detectable increases or decreases in the classification bias towards the Eff image the course of the session (Fig. 4A). One might expect, therefore, that the relationship between the AS response and behavioral bias (Fig. 3A and B) appearing at the beginning of the session remained stable over the course of the session. Were the neural responses also stable over the course of the session? If the neural responses depended on the interpretation of the image, we might be able to see evidence that the monkey's interpretation influenced the neural responses, pushing responses to the AS image in the direction of the monkeys’ choices with those stimuli. To consider this possibility we examined the relationship between the SI and the proportion of choices made with the AS as a function of trials in the session (Fig. 5A). Figure 5A shows the same relationship [*P*(AS bias to Eff) vs. SI] as Fig. 3A, but for four blocks in the session, consisting of the first–fourth blocks of trials of presentation of the AS image over the course of the session. These AS image trials were intermixed with other trials, including ones for which correct choices were available. The behavior was calculated from the entire session because there were no detectable changes in the behavior over the course of the session (and thus is repeated on the *x* axis in panels 1–4 in Fig. 5A). The left-most panel shows the relationship between the proportion of choices of the Eff stimulus and the SI during the first quarter block of trials of presentation of the AS. During this block, the correlation between the SI and average choice was low (*r* = 0.1411) and not significant (*P* = 0.2759). In each successive quarter block the correlation between the SI and the average choice increased, reaching a maximum of *r* = 0.4341, *P* = 0.0003 (Fig. 5A, right-most panel). The increase in the correlation coefficient can be seen in Fig. 5B, which plots the *r*-value as a function of the trial block.

The increase in correlation between the response to the AS and its interpretation (Fig. 5A and B) results from a difference in the response trends for the normalized response to the AS (SI) over the course of the session (Fig. 5A and B). The normalized response to the AS (SI) is shown for cells recorded in sessions in which the AS was more frequently classified as the Eff (solid line) or the Ineff (dashed line; Fig. 5C). As might be expected from the trends shown in Fig. 5A, in sessions in which the monkey frequently classified the AS as the Eff stimulus, the SI increased over the course of the session, reflecting a greater resemblance of the response to the AS to the response to the Eff stimulus. In sessions in which the AS image was frequently classified as the Ineff stimulus the SI decreased over the course of the session, reflecting increasing similarity of the response of the AS to the response to the Ineff stimulus. Thus, over the course of the session, the relationships of the AS to the Eff and Ineff images were modulated, during the monkeys’ consistent behavior, so that the neural responses to AS resembled the choices made by the animal about the AS image. (block 1 AS bias to Eff vs. AS bias to Ineff, *P* = 0.89; block 2 AS bias to Eff vs. AS bias to Ineff, *P* = 0.03). The AS response was normalized to the response to the Eff and Ineff stimuli. Therefore, the trends seen in the SI (Fig. 5C) could result from shifts in the response to any of the three stimuli. When neural responses were averaged (as opposed to the normalized response to the AS shown in Fig. 5C), the large variability in individual cell's responses masked the strength of the trends. The trends visible in the mean responses of the neurons showed that, in the Eff stimulus bias sessions, the response to the Eff stimulus decreased (∼2.98 spikes/s) and the response to the AS increased (∼2.67 spikes/s) over the course of the session, while the response to the Ineff stimulus remained constant. This had the effect of bringing the normalized response of the AS closer to the response to the Eff image. In Ineff image bias sessions, the response to the Ineff image increased slightly (0.723 spikes/s) and the response to the AS decreased slightly (0.522 spikes/s), bringing the response to the AS closer to the response to the Ineff. The trends became significant when individual cells were normalized to the response range for each cell.

Response modulation of the AS did not primarily reflect the ongoing variations in stimulus performance, as demonstrated by the fact that the better correlation between SI and average choice in a session occurred when average choice was calculated over the whole session, not within individual blocks of trials. The SI was still correlated with average trials in smaller blocks of trials (and also increased over the course of the session), but the *r*-values were slightly lower (0.3645 in block 4, *P* = 0.0379). A lower correlation is not surprising because of greater variability in calculating the performance in smaller blocks of trials. However, if the neural responses were wholly or largely determining the choices made with the AS, a greater correlation might be expected when the more local performance was compared to the neural responses in spite of the increased variability. This relationship was not seen.

No systematic changes in behavior with the AS image were expected over the course of a session, because the monkey was rewarded randomly for choices made about that image. Though no significant trends in choices of Eff or Ineff were present, the variability of choices over different sessions did decrease slightly over the course of the session (Fig. 4C). The small decrease in the variability in behavior over the course of the session suggests that the monkey might have realized (at least partially) that choices made about the AS image were not rewarded, and ceased using the interpretation of the image to drive choices. Thus, the classification of the AS in the early part of the session might be a better measure of the monkeys’ interpretation of the AS than the classification of the AS later in the session. If so, the interpretation of the AS at the beginning of the session might be even better correlated with the neuronal response to the AS than is the interpretation over the course of the entire session. To test this possibility, we calculated the correlation between the response to the AS image and the classification of the AS in the first block in the session (Fig. 5D). This relationship was particularly strong (*r* = 0.6013, *P* < 0.000001). The response of single neurons explains 36% of the variance in the interpretation of the AS images measured in the initial trials of the session.

The neural response modulation shown in this report did not result from a presaccadic modulation of the planned saccade to a visible target image. The monkeys did not make their behavioral report of choice (the saccade) over the period that neural responses are analyzed in this report (−500 to 1000 ms after the sample stimulus appears). The choice images did not appear until after this period. During the epochs of neural activity examined in this study, the monkeys maintained fixation on the sample stimulus and only made their choice much later, from targets that did not appear until the end of the epochs analyzed. The monkeys were trained to delay the choice of the matching stimulus. Therefore, no reaction times during the decision process are available in these data. The average time before picking the match from the choice array (which occurred at least 1400 ms after the onset of the sample image) did not change over the course of the session [mean = 860 ms after onset of the choice array; no significant trends (*P* > 0.05)]. Thus there were no detectable trends in the latency to responding over the course of the session.

### Correlation between interpretation and response to AS for individual images

**Fig. 6:**
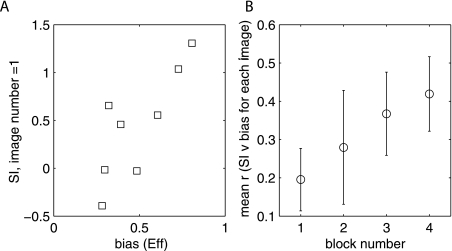
(A) SI for individual neurons recorded with image no. 1 (horse/giraffe) as a function average bias towards the Eff image in the session (*r* = 0.81, *P* = 0.02). (B) Mean correlation (*r*) between SI and stimulus bias in a session, averaged across all stimuli for which there were at least three available sessions, in blocks 1–4 of the session (*n* = 10 images, *p* of correlation different from zero, in block 4, *P* = 0.0019). The mean correlation increased over the course of the session: *r* = 0.99, *P* = 0.0059.

Individual AS images could also be classified with different degrees of bias towards the Eff and Ineff image across different sessions, correlating perhaps with the strength of the interpretation during these different sessions. Was the response to the identical AS image correlated with the degree of bias in behavior with that AS across different sessions? An example of positive correlation between classification bias of the AS in a session and the SI of a recorded neuron is shown for the images shown in Fig. 2 (the horse and giraffe). Selective cells were recorded in eight different sessions with this stimulus pair. Across these eight sessions, the monkey classified the same AS (the horse/giraffe, image pair 1, Fig. 1B) differently during different sessions. The normalized response (SI) to the AS (at the end of the session, block 4) was correlated with the bias in classification during the session, even for the identical stimulus (Fig. 6A; *r* = 0.81, *P* = 0.02). Across the population of stimuli, the mean correlation between bias in classification and the SI for each individual AS (*n* = 10 images with at least three different sessions in which a selective neuron was recorded) was significantly different from 0 by the end of the session (block 4, mean *r* = 0.42, *t*-test, different from 0, *P* = 0.0019). The mean correlation between bias in classification with a particular AS during a session and the normalized response to that AS increased over the course of the recording session (Fig. 6B). In this figure we have plotted the mean, across images, for the correlation between each AS image and the bias with that image in blocks 1–4 of the session. The responses for each individual AS image became aligned with the choices made about that stimulus based on the animal's interpretation (*r* = 0.99 over blocks, *P* = 0.0059).

### Time course of SI vs. bias relationship

**Fig. 7:**
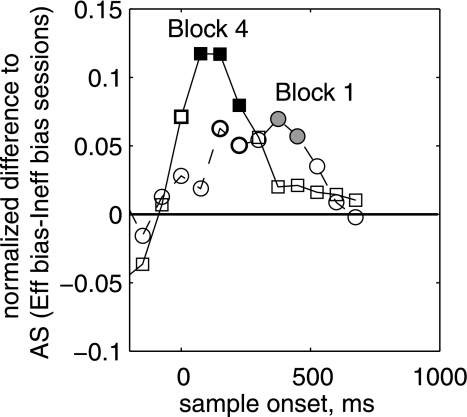
Mean normalized response to the AS in sessions in which the classification of the AS was biased towards the Eff image [*P*(bias to Eff) > 0.5] minus the response to the AS in sessions in which classification of the AS was biased towards the Ineff image [*P* (bias to Ineff) < 0.5] as a function of time; 300-ms epochs, starting at −225 ms before the onset of the AS. Dashed line, first quarter block of session; solid line, fourth quarter block of session. The difference in response peaked in the sample epoch 75–375 ms late in the session, and peaked in the epoch 375–675 ms after sample onset earlier in the session.

Neural responses to the AS shifted over the course of the session in favor of the choices made by the monkey. Did the response difference in different sessions appear at the onset of the visual stimulus or did it appear later during the response to the image or in the period after the presentation of the stimulus? A response difference that arose only after the image was presented would be compatible with the interpretation of the stimulus influencing the response in IT. Furthermore, the neural responses changed over the course of the session. Did the time course of the response difference in sessions with Eff and Ineff biases also change over the course of the session? The data shown above examined responses during the epoch of sample presentation, 75–375 ms after stimulus onset. Early in the session, across the sample epoch (75–375 ms after sample onset), there was no significant difference between the responses to the AS image in sessions in which AS was classified as the Eff (Eff sessions) and the Ineff sessions (*P* = 0.6292). Later in the session, the response difference to the AS between Eff and Ineff sessions during the sample period became significantly different (*P* = 0.0497). Did the time course of this response difference change over the course of the session? To examine this possibility, we calculated the mean response difference between the normalized response to AS image (see Methods) in Eff bias and Ineff bias sessions for different epochs, all 300 ms in length, but starting at different times before (or after) the presentation of the sample stimulus (Fig. 7). The response difference rose faster and peaked earlier during the block of trials collected at the end of the session (solid line) than during those trials collected at the beginning of the session (dashed line). The peak difference in the response to the AS image in Eff vs. Ineff bias sessions occurred during the presentation of the sample, when the difference first reached significance (Fig. 7, solid line, 75–375 ms after sample onset; *P* = 0.0497, unpaired *t*-test) during the last block of the session. However, the peak occurred later in the response (375–675 ms after sample onset) in the early trials, first reaching significance shortly after the end of the sample presentation (Fig. 7, dashed line, 450–750 ms after sample onset; *P* = 0.0492, unpaired *t*-test). The time course differences over the course of the session were compatible with an interpretation according to which the monkeys’ intrinsic bias (interpretation of the image) affected responses shortly after the AS presented in the initial trials (an ‘aha’ moment) but then modulated the response to the image itself.

### Response differences to nonambiguous images

**Fig. 8:**
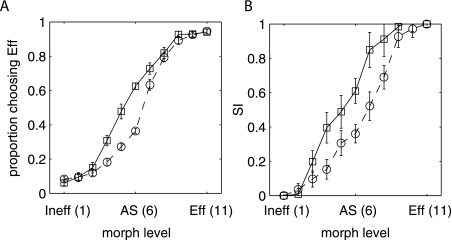
(A) Behavioral bias for all stimuli in sessions with Eff image bias (solid line, squares) and Ineff image (dashed line, circles) bias plotted as in Fig. 1C (as a function of stimulus level). (B) SI for all stimuli in sessions with Eff image bias (solid line, squares) and Ineff image (solid line, circles) plotted as a function of morph level.

This response differences to the AS images seen during Eff and Ineff stimulus bias also transferred to nearby, nonambiguous images that resemble the ambiguous image (Fig. 8A and B). When sessions were separated based on the behavioral bias with the AS image, the behavioral bias towards the Eff image bias was also present for the other images except for the easiest images, in which all bias disappeared (Fig. 8A). The neural response difference that paralleled the behavioral difference for the AS image also transferred to the nearby images. This transfer was visible when the SI was calculated for each of the different images, using the same calculation used to normalize the response to the AS image (Fig. 8B). The SI is defined as 0 for the Ineff image and 1 for the Eff image, and intermediate images showed SI values intermediate between the two alternatives. More notably, the bias in neural responses seen for the AS images was also present for other nearby images and did not disappear except for stimuli that most closely resembled the target images (morph levels 1–3 and 9–11).

### Choice probability

**Fig. 9:**
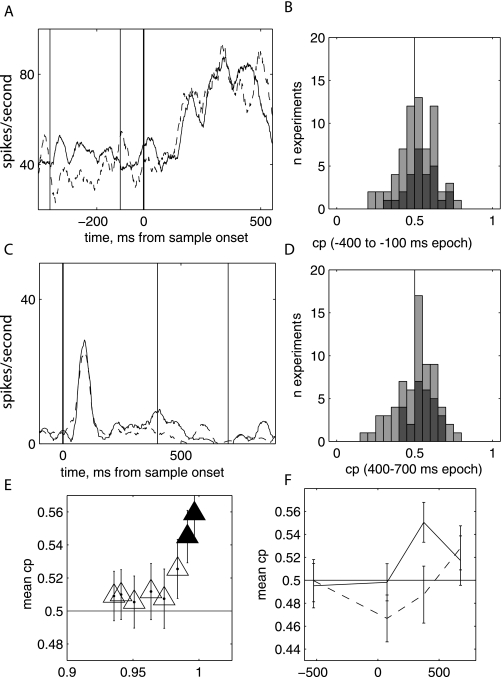
(A) Example neuron showing a difference in response during baseline period for trials in which the Eff image was chosen compared to those in which the Ineff image was chosen. (B) Distribution of choice probability calculated from baseline epoch. Dark grey bars, bias to Eff image sessions; light grey bars (*n* = 27), bias to Ineff image sessions (*n* = 37). (C) Example neuron showing a difference in response during the period shortly after the end of the stimulus for trials in which the Eff image was chosen compared to those in which the Ineff image was chosen. (D) Distribution of choice probability calculated from the post-sample epoch. Dark grey bars, sessions in which performance for Eff and Ineff images was > 0.96 (*n* = 27); light gray bars, other sessions. (E) Mean CP in post-sample epoch as a function of the mean behavior for the Eff and Ineff sample images. Each point represents the mean of the CP for all sessions in which the behavior was better than the value shown on the *x*-axis. (F) Mean choice probability in three different epochs for population of sessions in which performance was excellent (performance for easiest stimuli > 0.96; *n* = 26, solid line) and average performance (performance for easiest stimuli < 0.96, solid line) for four different epochs with respect to the onset of the sample stimulus at 0 ms. The *x*-axis shows the start of the sample epoch. When performance was excellent, choice probability was significantly greater than chance in the 375–675 ms epoch.

Another important relationship might be observable in the data: a relationship between trial-by-trial variation in the classification of the AS image and the neural response to that stimulus. This relationship is commonly calculated as a choice probability (CP) by sorting responses to the AS image based on the choice made about that stimulus in a particular trial. In these data, nonchance CP was visible but not robust or consistent. The analysis of CP showed properties consistent with interpretation and behavioral choices influencing the neural response, rather than neural response driving the choice. CP significantly different from chance could be seen in two different time periods. The first was in the period before the onset of the visual stimulus (the −400 to 100 ms epoch before the AS appeared; Fig. 9A and B). A representative cell with a significant difference in response between trials in which the Eff and Ineff images were chosen is shown for a session in which the overall bias was in favor of the Eff image (Fig. 9A). In this period, across the population, the mean CP was significantly different from chance in those sessions in which the monkey was biased towards picking the Eff image (Fig. 9B, dark grey bars; CP = 0.5443, *P* = 0.0238, *n* = 27). In the same period, the mean CP was not significantly different from chance in those sessions in which the monkey was biased towards picking the Ineff image (Fig. 9B, light grey bars; CP = 0.4974, *P* = 0.9054, *n* = 37). Thus, neural responses during the baseline period reflect the choice the monkey would make on a given trial when the stimulus was ambiguous. The lack of a CP significantly different from chance for the sessions in which the behavior was biased towards the Ineff stimulus suggests that the cells play a different role in the population in sessions in which the monkey was biased in favor of the stimulus preferred by the neuron vs. those in which the monkey was biased away from that stimulus.

CP returned to chance immediately after the presentation of the stimulus. During the epoch immediately after the transient response, CP was again significantly different from chance for those sessions in which the monkey performed well on the nonambiguous trials (performance for easiest trials > 96%; top two quintiles of behavior). An example cell showing this pattern of response is shown in Fig. 9C. In these sessions, in an epoch recorded during the trailing part of the response the population CP was significantly different from chance (Fig. 9D, 400–700 ms after sample onset; CP = 0.5335, *P* = 0.035, *n* = 27). The mean CP across the population was only significantly different from chance for the subset of sessions in which the performance was very good for the easiest trials, and decreased as sessions including poorer peak performance were included (Fig. 9E). The time course of CP is shown in 300-ms bins for the population of sessions in which performance was excellent (*n* = 27, solid line) and for all other sessions (*n* = 38; dashed line, Fig. 9F).

## Discussion

### Summary of main results

During the interpretation of ambiguous images, neural responses of individual cells in IT to ambiguous photographs (AS) were significantly correlated with the interpretation of the AS image (Fig. 3A). The correlation between interpretation and neural responses to the AS image was present for identical AS images that were classified differently during different sessions (Fig. 3B). The classification of the AS was uncorrelated with neural responses during the beginning of the session (Fig. 5A), but neural responses to the AS were driven in the direction of the biased choice made over the entire session over the course of the session (Fig. 5C). As a result of this shift in neural responses, by the end of each session, across sessions, there was a robust correlation between the classification bias in that session and the normalized responses of individual cells to the AS image (Fig. 5A and B). The changes in the relationship between neural response and interpretation occurred in the absence of any detectable changes in behavior (Fig. 4). Our study provides novel evidence for flexible coding of stimuli in IT, coding that can be modulated by the interpretation of images so that neural responses match the perception of stimuli by the monkey. These data suggest that IT is influenced by biases and processing of ambiguous images: what we think we see becomes what IT ‘sees’. The mechanism for these changes could be modulation by top-down circuits that control attention to feature (Bar, 2003, 2007; Ranganath *et al.*, 2004a; Ranganath & D’Esposito, 2005; Sergent *et al.*, 2005; Del Cul *et al.*, 2007; Furl *et al.*, 2007b) and space or a form of experience-dependent (but not learning-dependent) stimulus–stimulus association (Messinger *et al.*, 2001). The modulation of neural responses in IT, furthermore, might be induced by even arbitrary (as opposed to instructed or learned) biases in the perceptual experience of stimuli.

### Implications for neural coding in IT

Neural responses to stimuli in IT are often thought to depend on characteristic features contained within an image (Hung *et al.*, 2005; Kiani *et al.*, 2005, 2007; Leopold *et al.*, 2006). Our data show that the responses of individual cells in IT were correlated with the average classification of the stimulus. The first relationship demonstrated (Fig. 3A) is compatible with a feature-based explanation of IT neural responses. Conventional, feature-selective interpretation of the relationship in Fig. 4A would propose that an unknown feature is present in image A, and absent in image B, and the AS image contains a degraded version of that feature. If animals use that same unknown feature to drive the interpretation of the AS, a relationship between the proportional response to that feature and behavior (Fig. 3A) might be seen. Such an explanation of the data would still show a remarkable relationship between neural responses and the interpretation of the image: ∼36% of the variance in the initial interpretation of images, as reflected in the classification task, would be explained by the response of the single neurons (Fig. 5D).

However, the data also showed that responses to the identical image could be different, based on how they were classified during the course of the session (Fig. 3B), and that the relationship between behavior and the response to the AS image changed over the course of the session to match the interpretation of the image (Fig. 5). Furthermore, the responses of cells to identical visual stimuli were correlated with the differing bias in classifying those stimuli over different sessions (Fig. 6). These additional characteristics in the data pose additional challenges to a fixed-feature representation driving selectivity and behavior for the images and producing the correlation between neural and behavioral response seen in Fig. 3A.

Some form of feature-based attention (Moran & Desimone, 1985; Saenz *et al.*, 2002, 2003; Reynolds & Chelazzi, 2004; Hayden & Gallant, 2005; Maunsell & Treue, 2006; Serences & Boynton, 2007) combined with perceptual learning (Gilbert & Sigman, 2007) may explain the additional relationship between neural responses and behavior. Given that the recorded IT neuron responds to a target feature contained within the image, the monkey's average classification of the AS, the interpretation of the AS, reflects attention (or lack of attention) to that feature. Initially, that allocation of attention affected responses to the stimulus after the transient response to the stimulus and during the delay period (Fig. 7). As the image was repeated through the session, attention to that feature modulated neural responses during the representation of the image itself. The net result of attentional influence on the neural response might be modulation of activity in IT so that population activity across the cortex confirms and reinforces the interpretation of the image by the monkey. Attention to a feature could produce a relationship between selections made with the AS and neural responses by turning off (or suppressing) the responses of neurons that prefer unattended (and unused) features in the image while enhancing the responses of neurons that prefer the attended (and used) features of the image. Through this process, neural responses in IT become aligned with interpretation over the course of a session, suggesting that under the right circumstances (namely, well-trained behavior and the presence of stimulus ambiguity) the neural responses in IT can be modulated to match the perceptual experience of the monkey (Leopold, 2003; Kourtzi & DiCarlo, 2006; Sterzer & Kleinschmidt, 2007).

### Stimulus–stimulus associations as a mechanism for altering neural responses

Another possible mechanism for the response modulations is a form of stimulus–stimulus association, resulting from the biased performance of the task. The characteristics of the particular 2AFC-DMS task used in this report resembles that used in several examinations of association learning in IT (Miyashita, 1988; Messinger *et al.*, 2001). Over a period of time (Miyashita, 1988) or within a single session (Messinger *et al.*, 2001), neural responses to a sample image can begin to resemble the neural response to its paired associate as the monkey learns the associated relationship. In the 2AFC-DMS task used here the data are compatible with some form of association developing between the response to the morphed sample image and the choices associated with it. In this example, however, the association occurs as a result of task performance (or biased perception or interpretation) independently of any learning. No learning was possible because choices with the AS image were rewarded randomly, but the shift in neural responses might be compatible with similar shifts seen during association learning (Messinger *et al.*, 2001).

The monkey's interpretation of the AS results in biased performance which in turn results in a different set of stimulus experiences when behavior with the AS images was biased towards one choice or the other: the monkey views one of the choice stimuli more frequently. The resulting difference in stimulus experience could produce modulation of the neural response to the AS through passive temporal associations (Erickson & Desimone, 1999). To examine the passive performance of the task (as opposed to the intrinsic bias chosen by the monkey), neural responses could be examined when a monkey is trained to produce biased classification of the stimuli (through rewarding ambiguous images differently, for example) and compared to the responses obtained when the biases were voluntary (Suzuki & Grabowecky, 2007). Such training might not necessarily influence the properties of the neurons if the training did not change the interpretation of the image, but instead changed the rule used to classify the images (Muhammad *et al.*, 2006).

### Time course of difference in response during different interpretations of the AS

The time course of the difference in responses to sessions with different biases in the classification of AS images also concurs with a recurrent processing scheme for this multistep modification of neural responses in IT (Ranganath *et al.*, 2004b; Nobre *et al.*, 2006; Philiastides & Sajda, 2006; Philiastides *et al.*, 2006; Del Cul *et al.*, 2007; Furl *et al.*, 2007a). Early in the session, differences in neuronal response between sessions that resulted in a interpretation of the AS as one image vs. the other appeared late in the response to the sample. Later in the session the response difference appeared at the onset of the image (Fig. 7). The time course is compatible with a top-down, late-arriving signal producing response differences in the two types of sessions. As the session progressed, the late-arriving signal eventually modulated the response to the stimulus itself, producing a response difference to the image at the onset of the response to the sample (Gilbert & Sigman, 2007). In addition, the time course showed a process that occurred gradually as opposed to the more rapid time course commonly attributed to attention.

### Interpretation of choice probability

The main results in this report show a relationship between the mean response to a stimulus and the mean behavior with that stimulus, and a change in that relationship over the course of a session in which behavioral classification remained consistent. A related but different question is the relationship between trial-by-trial variations in the firing rate of a neuron and the trial-by-trial variations in choices, calculated as choice probabilities (Britten *et al.*, 1996; Parker *et al.*, 2002; Uka & DeAngelis, 2003; Liu & Newsome, 2005; Purushothaman & Bradley, 2005; Uka *et al.*, 2005). The presence of choice probability significantly different from chance can be interpreted as supporting a critical role for the recorded neuron in the decision making process or as a critical influence of the decision on the response of the neuron (Britten *et al.*, 1996; Parker *et al.*, 2002; Leopold, 2003). Choice probability significantly different from chance was only found in limited instances within this data set. The limitations might stem from an incomplete control of the behavior by the sample presented during the stimulus period (Fig. 4C–E). The monkey may have realized over the course of the session that ambiguous images would not result in reward, modifying behavior to reflect that knowledge. Under these conditions, the choice made at the end of the trial in the task is not perfectly correlated with the perception or interpretation of the image during the sample period. Choice probability can only be interpreted when the choice on individual trials is tightly correlated with the perception of the stimulus; thus the interpretation of the CP in these data must be approached with some caution.

Our data showed two instances in which choice probability across the population was significantly different from chance. The first was in a period before the onset of the AS, during the baseline period, for those sessions in which the monkey was biased towards choosing a stimulus that drove a good response in the recorded cell (bias towards Eff). The response during the baseline period can be interpreted as the neural signature of a bias in the monkey's expectations about the stimulus, which subsequently influence the choices made by the monkey (Chelazzi *et al.*, 1993). On first glance, nonchance CP during this period might seem impossible: the monkey could not know that the stimulus was going to be ambiguous before it appeared. However, this improbability can be reconciled by considering that the stimulus response influenced the choice made on nonambiguous trials. Therefore, it is only in ambiguous trials that the activity during the baseline has a significant impact on choice; in non-ambiguous trials, the responses to the sample diminishes any pre-existing bias in the neural responses (Serences & Boynton, 2007).

The second instance of choice probability significantly different from chance occurred in the period immediately after the presentation of the sample stimulus, for those sessions in which the monkey performed well on the easiest sample stimuli. These data suggest that, when the monkey was performing very well and ‘lapse’ errors (consisting of errors driven by factors other than the stimulus) are low, the choice of the monkey is significantly linked to neural responses in the period after the sample presentation. This timing concurs with the timing found in the electroencephalographic and magnetoencephalographic signas during the classification of ambiguous images (Sergent *et al.*, 2005; Del Cul *et al.*, 2007; Furl *et al.*, 2007b). The timing, further, argues for an interpretation of choice probability that results from the decision made about the stimulus, rather than primarily producing the decision about the stimulus (Britten *et al.*, 1996).

### Implications for the interpretation of neural responses to ambiguous stimuli

Neural responses in this task were modulated independently of any detectable change in the behavior or learning over the course of the session. This pattern is compatible with the interpretation of the image driving the modification of neural responses and the data concur with experiments in human subjects that suggest that ambiguous stimuli are interpreted in the context of expected stimuli; expectations drive a bias, and a fronto-parietal network, that can influence neural responses in sensory areas (Bar, 2003, 2007; Ranganath *et al.*, 2004a; Ranganath & D’Esposito, 2005; Sergent *et al.*, 2005; Del Cul *et al.*, 2007; Furl *et al.*, 2007b).

In conclusion, these data provide evidence that neural responses in IT are modulated over the course of the session by the interpretation of ambiguous stimuli. The modulation may create patterns of cortical responses in IT cells that match the behavioral classification of individual stimuli by the monkey (Allred *et al.*, 2005; Liu & Jagadeesh, 2008).

## Abbreviations

2AFC-DMS: two-alternative forced-choice–delayed match-to-sample
AS: ambiguous sample
CP: choice probability
Eff: effective
Ineff: ineffective
IT: inferotemporal cortex
PSTH: peristimulus time histogram
SI: similarity index
